# Matrix Stiffness Induces Pericyte-Fibroblast Transition Through YAP Activation

**DOI:** 10.3389/fphar.2021.698275

**Published:** 2021-05-31

**Authors:** Feng Feng, Xueyan Feng, Di Zhang, Qilong Li, Li Yao

**Affiliations:** ^1^ State Key Laboratory for Structural Chemistry of Unstable and Stable Species, CAS Research/Education Center for Excellence in Molecular Sciences, Institute of Chemistry, Chinese Academy of Sciences, Beijing, China; ^2^ School of Chemical Sciences, University of Chinese Academy of Sciences, Beijing, China

**Keywords:** hydrogel, matrix stiffness, blood vessel, pericyte, fibroblast, tumor

## Abstract

Vascular pericytes, important mural cells that retain progenitor cell properties and protect vascular integrity in healthy tissues, are often associated with tumor development, but their functions in cancer invasion remain elusive. One prominent outcome of tumor occurrence is that the microenvironment of the lesion often stiffens, which could change resident cell behavior. Here, we found pericytes are matrix stiffness-responsive and mechanical stimuli induce pericyte-fibroblast transition (PFT). Soft PA gels that mimic the stiffness of healthy tissues retain the identity and behavior of pericytes, whereas stiff PA gels that reflect the stiffness of tumorous tissues promote PFT and the mobility and invasiveness of the cells. Matrix stiffness-induced PFT depends on the activation of YAP (Yes-associated protein), a transcription factor, which, upon receiving mechanical signals, transfers from cytoplasm to nucleus to mediate cell transcriptional activities. Our result reveals a mechanism through which vascular pericytes convert to fibroblasts and migrate away from vasculatures to help tumor development, and thus targeting matrix stiffness-induced PFT may offer a new perspective to the treatment of cancer metastasis.

## Introduction

Extracellular matrix (ECM) is non-cellular component that provides biochemical and structural supports for its cellular constituents (Walker et al., 2018). Rather than static and inertial filling, ECM is physiologically active, constantly under remodeling and responsible for numerous cellular activities, such as cell-cell communication, cell proliferation, and cell adhesion (Bonnans et al., 2014). *In vitro* experiments have shown that most animal cells maintain viable only when adhering to a substrate, and *in vivo* studies have demonstrated that cells rely heavily on interacting with the surrounding ECM to survive (Vogel and Sheetz, 2006). In healthy organs, the production and degradation of fibrous proteins in ECM are well orchestrated to maintain tensional homeostasis, which guarantees tissue structure and proper function. However, tumorigenesis and other pathological conditions often tilt the balance between ECM production and degradation, resulting in an increased quantity of fibrous proteins accumulation (Weis and Cheresh, 2011). The overabundance of fibrous proteins in the tumorous microenvironment crosslink and form mesh-like structures that resist compressive or tensile forces and strengthen in a strain-stiffening manner (Mohammadi and Sahai, 2018). Increased matrix stiffness of tumor ECM promotes tumor progression by interfering with cell–cell adhesion, cell migration, and ultimately the integrated mechanical machinery that translate extrinsic mechanical stimuli into global changes of cell functions (Chen, 2008; Liu et al., 2016).

Preclinical and clinical evidence shows that the aberrant ECM remodeling in tumor tissue is strongly correlated with an invasive and metastatic phenotype of most solid tumors. It has been reported that ECM stiffness enhances cell growth and migration (Lo et al., 2000), and the rigidity of the ECM dictates differentiation of mesenchymal stem cells (Engler et al., 2006; Chaudhuri et al., 2016). Though much is known about how biochemical signaling regulate cell behavior, relatively little is known about how the mechanical stiff microenvironment directs tumor development (DuFort et al., 2011). Yet whether the stiffness of tumor microenvironment promotes tumor progression through dictating behaviors of resident mesenchymal stem cells has not been fully understood.

Blood vessels are essential to tumor growth and progression. Without angiogenesis, tumors cannot grow beyond a very small size, nor can they metastasize to colonize distant organs (Bielenberg and Zetter, 2015). Once tumorigenesis occurs and the lesion reaches a few millimeters in depth, tumor cells start to exploit their surroundings to compensate for center nutrient and oxygen deprivation. Angiogenesis is one of the outcomes of the exploitation (Weis and Cheresh, 2011). Though angiogenesis may provide with more oxygen and nutrients, the ultimate result is that tumor vasculature is continuously getting remodeled and is characterized by high capillary densities, enlarged capillaries with more blood flow, and increased vessel leakiness (Lugano et al., 2020). These are all characteristics that facilitate component exchange, immune transmigration, as well as tumor dissemination. Overall, the formation of new blood vessels in tumor tissues create a host-hostile but tumor-friendly microenvironment that fuels tumor progression. Though much has been studied about the relationship between tumor vascular formation and stromal cells, the relationship between ECM stiffness and blood vessel abnormality has not been fully assessed.

Pericytes, mainly described as mural cells, are associated with vasculatures and protect their integrity (Krueger and Bechmann, 2010). However, whereas in healthy developmental tissues, appropriate numbers of pericytes are to stabilize vasculatures, prevent leakiness, and promote maturation (von Tell et al., 2006; Chakroborty et al., 2011). The pericytes surrounding tumor vessels are less abundant and develop abnormal phenotypes, including aberrant cell shape, changes in marker expression, and loose vessel attachment (Cooke et al., 2012). It is possible that mural cell deficiency contributes to the abnormal function of tumor vessels. Recent studies show that pericytes are tremendously plastic in their differentiation potential and can differentiate into different cells, including adipocytes, chondrocytes, osteoblasts, phagocytes, granulocytes, and skeletal muscle (Dellavalle et al., 2011; Mills et al., 2013; Tian et al., 2014). In the bone marrow, NG2+ pericytes in arterioles promote hematopoietic stem cell (HSC) quiescence and are important for HSC maintenance (Mendelson and Frenette, 2014). Under pathological conditions, pericytes are reported to differentiate into myofibroblasts, contributing to kidney fibrosis. Given that the rigidity of the ECM potently controls the differentiation of mesenchymal stem cells and the mechanical force between mesenchymal stem cells and their microenvironments acts as a molecular switch that determines cell fate, we ask whether ECM stiffness in tumor tissues affects the integrity of tumor blood vessels by inducing the differentiation of pericytes.

Here, by culturing pericytes on different polyacrylamide (PA) gels that mimic stiffness from healthy to pathological tissues, we found pericytes are matrix stiffness-responsive and mechanical stimuli induce pericyte-fibroblast transition (PFT). Soft PA gels that mimic the stiffness of healthy tissues retain the identity and behavior of pericytes, whereas stiff PA gels that reflect the stiffness of tumorous tissues promote PFT and the mobility and invasiveness of the cells. Matrix stiffness-induced PFT depends on the activation of YAP (Yes-associated protein), a transcription factor, which, upon receiving mechanical stimuli, transfers from cytoplasm to nucleus to mediate cell transcriptional activities. Our results reveal a mechanism through which vascular pericytes convert to fibroblasts and migrate away from vasculatures to help tumor development, and thus targeting matrix stiffness-induced PFT may offer a new perspective to the treatment of cancer.

## Materials and Methods

### Antibodies, Drugs, and Reagents

The primary antibodies used were: anti-FSP1 polyclonal antibody (07–2274, Millipore), anti-NG2 monoclonal antibody (ab83508, abcam), anti-αSMA monoclonal antibody (ab32575, abcam), anti-non-muscle myosin II monoclonal antibody (ab238131, abcam), anti-YAP1 monoclonal antibody (ab76252, abcam). TRITC phalloidin (CA1610) and DAPI (C0065) were purchased from Solarbio. The secondary antibodies used were: Alexa Fluor 488-labled Goat Anti-Rabbit IgG (H + L) (A0423), Alexa Fluor 488-labled Goat Anti-mouse IgG (H + L) (A0428), Alexa Fluor 647-labled Goat Anti-rabbit IgG (H + L) (A0468) and Alexa Fluor 647-labled Goat Anti-mouse IgG (H + L) (A0473). The secondary antibodies were all purchased from Beyotime. Matrigel (356,234, Corning) was purchased from Corning and used according to the manufacturer’s instruction. Collagen, Verteporfin, acrylamide, bis-acrylamide, ammonium persulphate, tetramethylethylenediamine, were purchased from Sigma-Aldrich. Ferric acetylacetonate [Fe (acac)_3_], oleic acid (OA), oleylamine, 1-octadecene and dopamine hydrochloride were purchased from Aladdin. Ethanol, tetrahydrofuran (THF), and cyclohexane were obtained from Beijing Reagents Co., China.

### Pericyte Isolation and Maintenance

Pericyte isolation was conducted as previously described (Hosaka et al., 2016). Briefly, fresh mouse lungs were cut into small pieces by scissors, followed by incubation with Type I and II collagenase (each 1.5 mg/ml; Sigma) at 37°C for 40–60 min. Filtered single-cell suspensions were collected by centrifugation at 100 × g for 10 min. Pellets were incubated with an anti-NG2 antibody for 45 min on ice and then with a Cy5-labeled goat anti-mouse antibody (Beyotime) for 15 min on ice. Washed cells were further incubated with anti-Cy5 magnetic beads and beads’ positive fractions were collected using magnetic columns. Purity of positive fractions was confirmed using FACSort and CellQuest software (BD Bioscience). Fe_3_O_4_ nanoparticle fabrication and hydrophilization. Isolated pericytes were cultured with pericyte specific medium supplemented with 10% fetal bovine serum (gibco), 1% pericyte cytokine (Icell), and 1% Penicillin-Streptomycin solution (Solarbio). Pericyte isolation was conducted by iCell company in Shanghai China.

### Nanoparticle Fabrication and Hydrophilization

Fe_3_O_4_ nanoparticle was synthesized as previously described (Li et al., 2019). Briefly, Fe (acac)_3_ (2 mmol), oleic acid (6 mmol), oleylamine (6 mmol), and 1-octadecene (20 ml) were mixed and heated to 200°C for 30 min and the mixture was refluxed at 300°C for another 30 min before cooling to room temperature. The whole process was under nitrogen protection. The product was precipitated using ethanol and washed with ethanol/cyclohexane for three times before dispersed in cyclohexane. The surface of the nanoparticles was modified using dopamine.

### Cell Viability Assay

Cell viability was evaluated using Cell Counting Kit-8 (Solarbio) following the manufacturer’s protocols. Briefly, cells were seeded into 96-well plates at a density of 3,000 cells/well and treated with nanoparticles at different concentrations (0, 20, 30, and 50 ug/ml) for 24 h. Then 15 ul CCK-8 was added to each well and incubated for 2 h at 37°C. The optical density was measured at an absorbance of 450 nm using a microplate reader.

### Bis-acrylamide-PA Gel Fabrication

Polyacrylamide gel preparation was adapted from protocols described previously (Tse and Engler, 2010). Briefly, coverslips were rinsed and placed in plasm cleaner for two minutes. 200 ul APTES was added onto each coverslip for 5 min. The coverslips were rinsed again and 0.5% glutaraldehyde in PBS was added for 30 min before the coverslips were blew dry by nitrogen. Gel solution mixed from Acrylamide, bis-acrylamide, 1/100 APS, and 1/1000 TEMED was degassed and added onto each coverslip (150 ul per coverslip). Another batch of coverslips that have previously been treated with DCDMS were used to weight on the gel solution to ensure the gel solution forms a thin layer that were sandwiched in between two coverslips. Collagen was used to modify gel surface. The surface morphology and stiffness of the gel was studied using AFM.

### Immunostaining

After pericytes (or pericyte spheroids) were seeded on PA gels for 24 h. The cells were washed three times with PBS. 4% paraformaldehyde solution was used to fix pericytes for 10 min. Following pericyte fixation, PBS was used to wash pericytes for another three times and 0.1% Triton X-100 was used to permeate the cells for 5 min. After PBS washing, the cells were incubated with anti-NG2 (ab83508; abcam), anti–αSMA (ab32575; abcam), anti-FSP1 (07–2274; Millipore) at 4°C overnight. The cells were then washed with PBS and incubated with secondary antibodies for 1 h at room temperature. The cells were costained with Alexa Fluor–phalloidin and/or DAPI for 5 min at room temperature. Images were acquired using an FV10 Olympus confocal fluorescence microscope. Fluorescence intensity was analyzed using ImageJ.

### Wound Healing Assay

To assess cell migration, pericytes were cultured on collagen-modified PA gel plates with complete medium till 100% confluence. Then, the medium was removed and cells were washed. A wound was created by scratching cell layers in the middle. The cell layers were rinsed with PBS to remove dead cells and cultured with serum-free medium. The wounds were observed under a microscope and images were taken at previously appointed time. The images were analyzed using ImageJ.

### Transwell Invasion Assay

Pericytes (2 × 10^4^ cells in 150 μl of serum-free medium) were trypsinized from different matrices and loaded into the Matrigel (Corning) -coated upper chamber of the Transwell; the lower chamber was loaded with 500 μl complete medium. 3 h later, A549 lung cancer cells (4 × 10^4^ cells in 150 μl of serum-free medium) were added on the surface of the pericyte layer in the upper chamber. After culturing at 37 C for another 12 h, the cells that grew on the upper membrane were wiped with a cotton swab while the cells that had migrated through the membrane and grew on the lower membrane were fixed with 4% paraformaldehyde and stained with Trypan blue. The stained cells on the lower membrane were observed under a microscope and at least five visual fields were analyzed for each group.

### Tumor Sphere Culturing

Tumor sphere culturing assay was conducted as previously described (Wang et al., 2020). Briefly, 96-well plates were pre-coated with 20 μl of a 2% low-melting-temperature agarose. Pericytes were seeded at a density of 2 × 10^3^ cells/well and cultured in a 37°C, 5% CO_2_ humidified incubator. Following their formation, pericyte spheroids were transferred on collagen-coated PA gel plates and cultured at 37°C to allow pericyte invasion. 2 days later, the plates were put under a microscope and pictures were taken.

### FIRMS Measurement

FIRMs measurement was conducted as previously described (Yu et al., 2019; Xu et al., 2020). Briefly, pericytes were seeded in FIRMs slices and labeled with Fe_3_O_4_ nanoparticles. The slices were rinsed with PBS to remove dead cells and uninternalized nanoparticles. Following 2 min magnetization of nanoparticle-labeled pericytes, the slice was mounted onto FIRMS and the initial remanence signal was recorded. The slices were then subjected to a series of centrifugal forces that increased stepwise, and each time after force application, the slices were taken back to FIRMS to record the remanence again. The centrifugal force applied to the cells was calculated as: F = m·ω^2^·r, where F is the relative centrifugal force, m is the buoyant mass, ω is the angular speed, and r is the distance from the sample to the center of the centrifuge. The relative adhesion force was defined as the centrifugal force corresponding to the half maximum signal.

### *In vitro* Assay of Angiogenesis

*In vitro* assay of angiogenesis was conducted as previously described (McGonigle and Shifrin, 2008). Pericytes between passage one and six were used to obtain a good capillary network. Briefly, Matrigel thawed at 4°C was pipetted onto different PA gels (500 ul/gel) and incubated at 37°C for 30 min to allow Matrigel to set. 0.5 ml resuspended pericytes at a concentration of 1.4 × 10^5^ cells/ml was added onto each Matrigel coated PA gels and cultured in a 37°C, 5% CO_2_ humidified incubator. 18 h later, the plates were put under microscope and phase pictures were taken with 10 × objective lens.

### Statistical Analysis

All data are presented as mean ± SEM. GraphPad Prism 8 was used for statistical analysis. Means of the two groups were compared using student *t*-test. The means of multiple groups were analyzed by one-way ANOVA. *p* < 0.05 indicates significant difference.

## Results

### Matrix Stiffness Changes Pericyte Morphology

**FIGURE 1:**
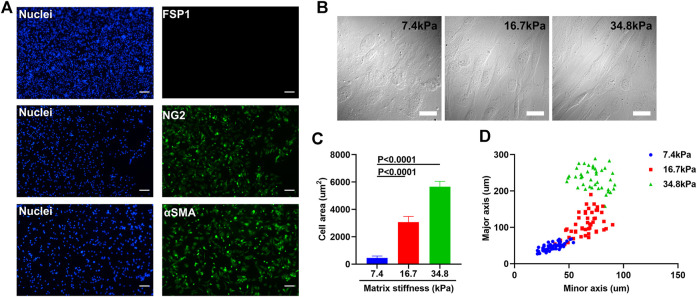
Matrix stiffness changes the morphology of pericytes isolated from mouse lung vasculatures. **(A)** Representative immunofluorescence images of pericytes that are NG2 and αSMA positive but FSP1 negative. Cell nuclei were stained with DAPI. Scale bar is 200 um. **(B)** Morphology changes of pericytes cultured on different matrices that vary in stiffness. Scale bar is 20 um. **(C)** Statistical analysis of the spreading areas of pericytes on different matrices. **(D)** The distribution of the major axis of pericytes vs. their minor axis on different matrices.

To investigate the possibility that tumor microenvironment might ruin the integrity of tumor blood vessels by inducing pericyte differentiation, we isolated primary pericytes and cultured them on substrates of different stiffness that reflect the rigidity of tissues ranging from pathological conditions to healthy ones. Though the ultrastructural characteristics of pericytes have been well-studied, pericytes remain a relatively mysterious cell type, with no specific markers enabling their clear-cut identification, because of the heterogeneous distributions and functions of pericytes in various tissues. Chondroitin sulfate proteoglycan4/neural glial antigen 2 (NG2) is expressed on the surface of pericytes during angiogenesis. Alpha smooth muscle actin (αSMA) (12) is among the contractile filaments of pericytes. Though these two markers are also expressed by other cell types, for example NG2 is expressed on glial precursor O_2_A cells and aSMA on smooth muscle cells, the combination of these two markers suffices to identify pericytes (Yamazaki and Mukouyama, 2018). As presented in Figure 1A, the pericytes isolated from mouse lung vasculature are NG2 and αSMA positive, which confirms their identity. The purity of the pericytes was confirmed by co-staining pericytes with FSP1, a fibroblast specific cell surface marker. The FSP1 negative and NG2 and αSMA positive results assures the identity and purity of pericytes. To study the influence of matrix stiffness on pericytes, we produced a series of PA substrates of different stiffness (Supplementary Figure S1A, B). By culturing pericytes on these substrates, we observed that stiffness-stimulated pericytes changed their morphologies along the substrate stiffness, which indicates that pericytes are matrix stiffness responsive and could potentially be influenced by the rigidity of surrounding environment (Figure 1B). Pericytes stretched larger as the substrate stiffness increased (Figure 1C) and formed elongated and spindle-like cell shapes (Figure 1D), which were very similar to fibroblasts. Given that some papers earlier have reported that in tumor microenvironment NG2+ pericyte numbers were decreased and fibroblasts were found to be enriched (Welen et al., 2009; Stallcup, 2013), we suspect that matrix stiffness could induce pericyte-fibroblast transition.

### Matrix Stiffness Increases the Adhesion Force of Pericyte

**FIGURE 2:**
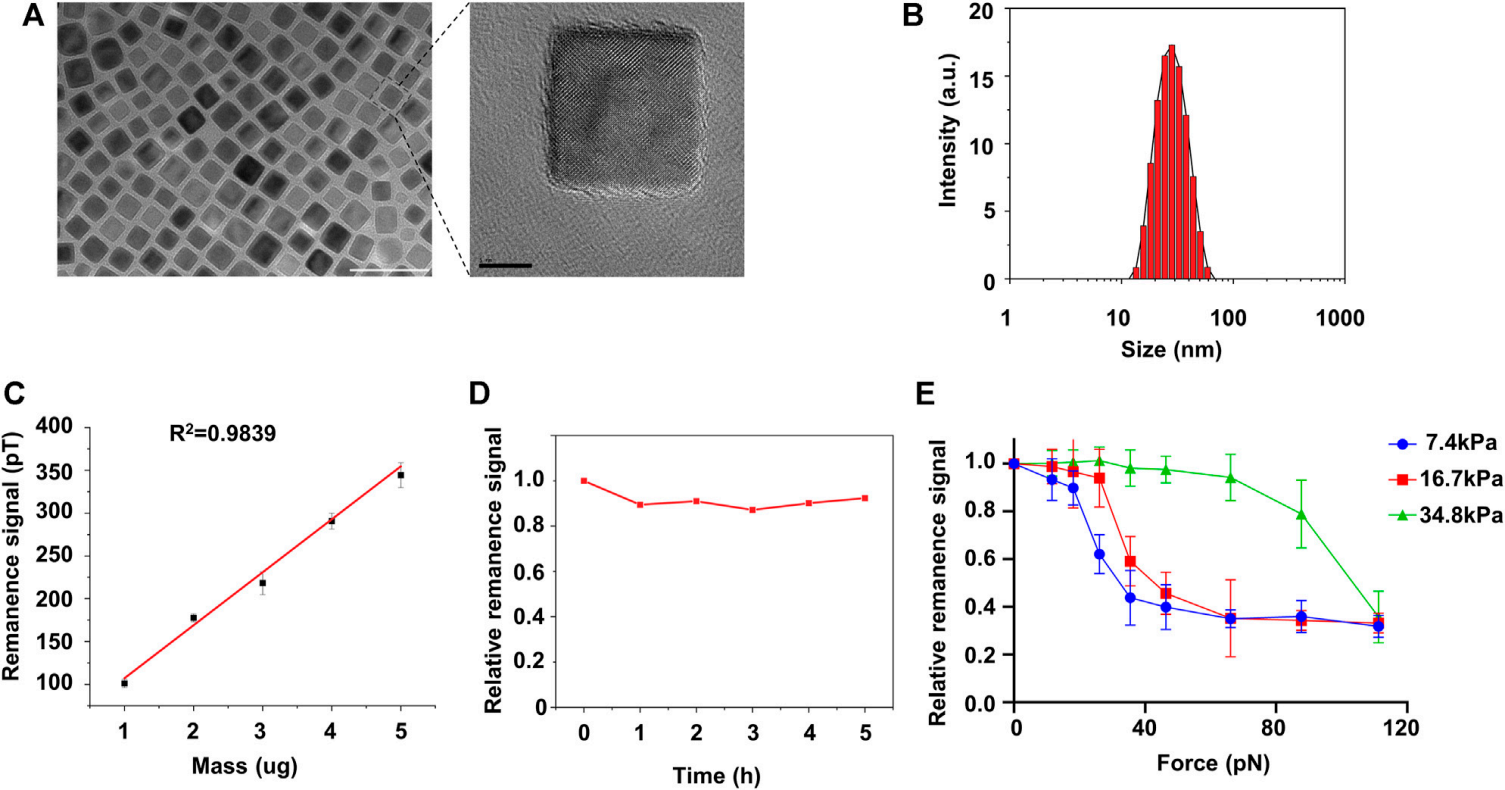
FIRMs measurement of pericyte adhesion force on different matrix. **(A)** Left: TEM images of Fe_4_O_3_ nanoparticles. Scale bar is 50 nm. Right: amplification image of the encircled nanoparticle in the left image. Scale bar is 5 nm **(B)** Size distribution of the nanoparticles post-hydrophilization **(C)** The lineal relationship between the remanence signal and their mass **(D)** Relative remanence signal of Fe_4_O_3_ nanoparticles vs. time. The concentration of the nanoparticle used here is 30 ug/ml. **(E)** Adhesion force of pericytes on different matrices measured by FIRMs.

Compared with pericyte, activated fibroblast interacts rigorously with its surroundings (Feng et al., 2019). To exam if matrix stiffness induces pericyte-fibroblast transition, we fabricated 20 nm Fe_3_O_4_ nanoparticles (Figure 2A) and studied pericyte adhesion force using the nanoparticles as probes in force-induced remanence magnetic microscopy (FIRMs). The nanoparticles were cubic and well-dispersed in aqueous solutions (Figure 2A). The nanoparticles have good biocompatibility (Supplementary Figure S2). Dynamic Light Scattering result shows good size homogeneity (Figure 2B). It should be noted that the nanoparticles used in FIRMs should retain high remanence magnetism, as FIRMs, by measuring forces between either molecules or cells, de facto measures the post-perturbation remanence signal of the nanoparticles. Thus, we studied the magnetic properties of the nanoparticles. Remanence signal study reveals good lineal relationship between the remanence signal and the mass of the nanoparticles (Figure 2C). The optimal concertation of the nanoparticles used in measuring pericyte adhesion force was determined to be 30 ug/ml. To exclude the possible that the remanence signal reduction measured by FIRMs was caused by the magnetic relaxation of the nanoparticles, rather than by external-perturbation induced remanence signal reduction, we analyzed the remanence signal of the nanoparticles against time. The result shows that the nanoparticles have high remanence signal retention ability, as 5 h after magnetization, the remanence signal of the nanoparticles remains fluctuating around its initial value (Figure 2D). Put together, these magnetism studies indicate that these nanoparticles are well-qualified probes to be used in FIRMs to measure pericyte adhesion force. 24 h after culturing on PA substrates of different stiffness, pericytes were transferred onto FIRMs slices and the adhesion force was measured (Figure 2E). On soft substrate (7.43 kPa) pericytes started to detach from FIRMs slices when the external perturbation force exceeds 20 pN, whereas on stiff substrates (34.88 kPa) they persisted their stable adhering even when the perturbation force exceeds 60 pN. The detachment force of pericytes cultured on substrates of medium stiffness (16.7 kPa) landed in between. Thus, we concluded that matrix stiffness increased the adhesion force of pericytes.

### Matrix Stiffness Increases the Invasiveness of Pericytes

**FIGURE 3:**
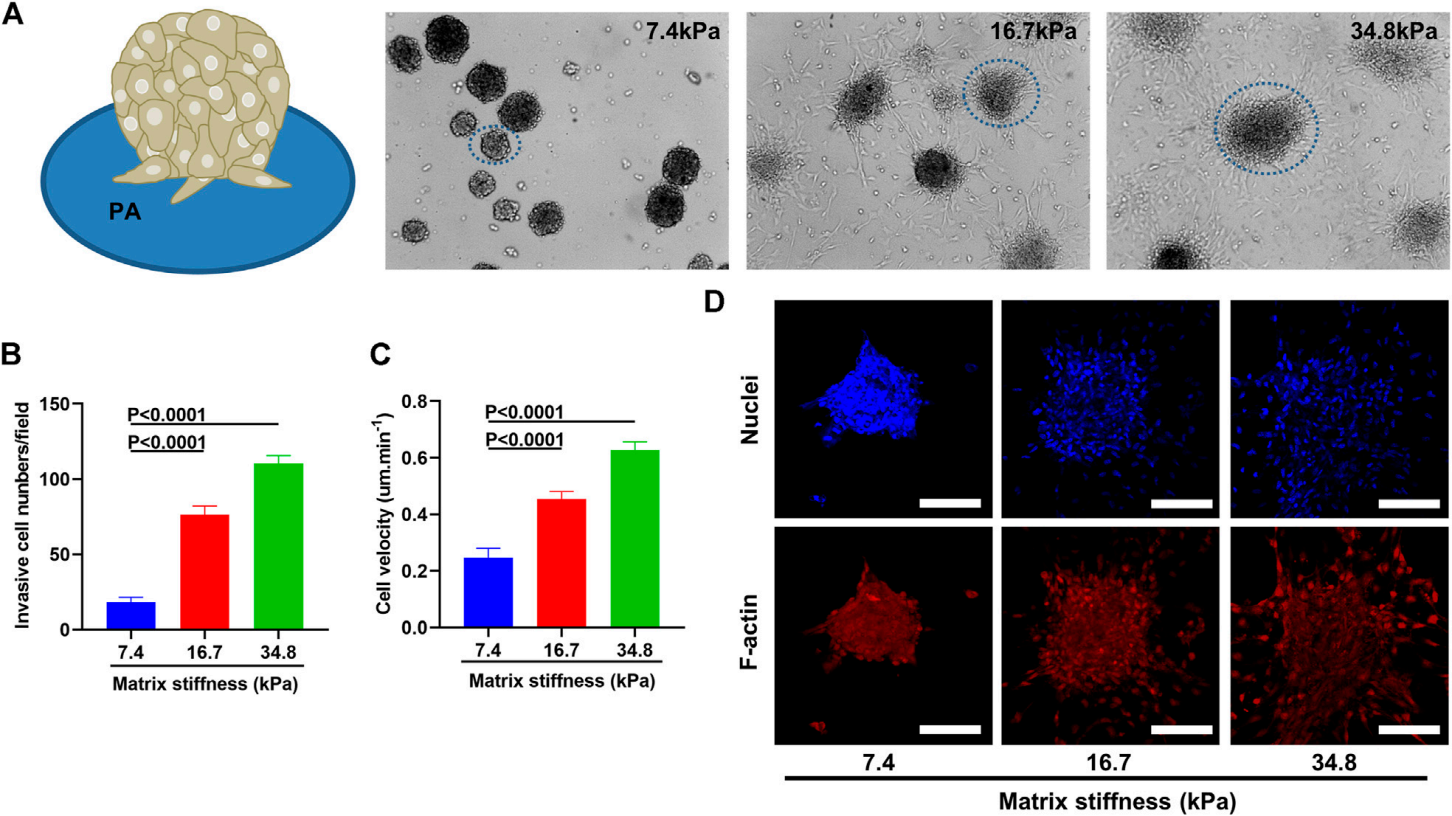
Matrix stiffness induces pericytes to migrate away from pericyte spheroid and invade their surroundings. **(A)** Pericyte spheroids cultured on collagen-modified PA gels. **(B)** Statistical analysis of invasive cell numbers of pericytes cultured on different matrices. **(C)** Migration velocity of pericytes cultured on different matrices as measured by wound healing assay. **(D)** Representative images of pericyte spheroids encircled in A. Cell nuclei were stained with DAPI. Scale bar is 90 um.

Considering that tumor associated fibroblasts when activated migrate away from tumor center and tend to move from softer to stiffer regions when cultured on a matrix-coated substrate (Feng et al., 2019), we wonder if matrix stiffness could enhance the invasiveness of pericytes. To address this question, we cultured pericytes into small spheres and transferred them onto PA gels of different stiffness (Figure 3A). 2 days after culturing, pericyte spheres on soft substrate (7.43 kPa) remained spherical and displayed a clear-cut edge with very sparse single cells invading the surrounding environment; however, on stiff substrate (34.88 kPa) pericyte spheres became larger and displayed a fuzzy edge with lots of single cells invading the surrounding environment. Pericyte spheres cultured on substrate of medium stiffness (16.7 kPa) displayed more invading single cells than those did on soft substrate but less than on stiff substrate (Figures 3B,D). To further analyze the influence that matrix stiffness had on pericyte invasiveness, we conducted wound healing assay (Supplementary Figure S3). As presented in Figure 3C, pericyte migration rate increases along with the increase of matrix stiffness. Taken together, these data demonstrate that matrix stiffness increases the invasive behavior of pericytes in both the invading cell numbers and their velocity.

**FIGURE 4:**
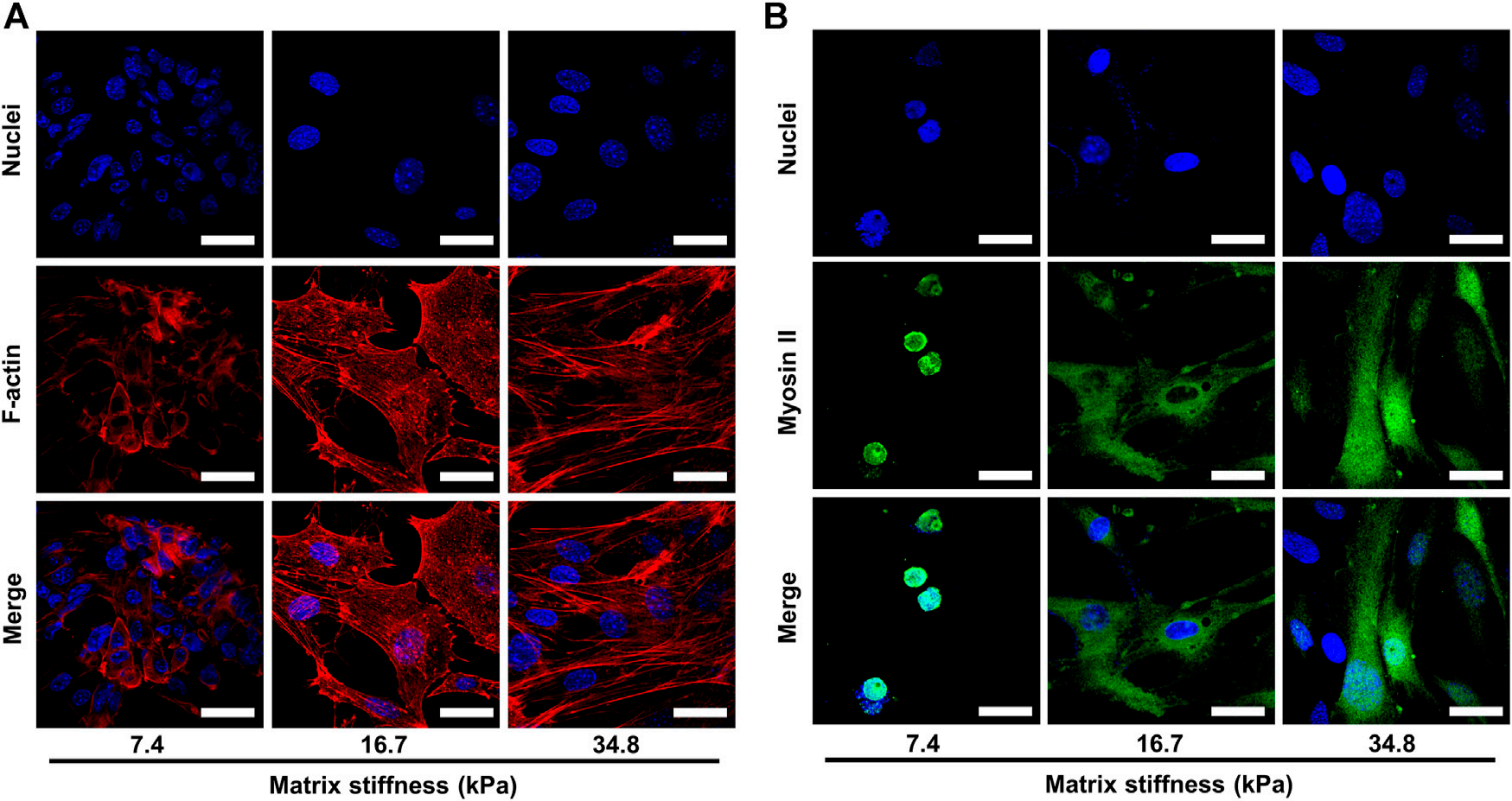
Representative images of the cytoskeletal elements of pericytes cultured on different matrices. Cell nuclei were stained with DAPI. **(A)** Representative images of F-actin assembling in pericytes on different matrices. Scale bar is 30 um. **(B)** Representative images of myosin II in pericytes on different matrices. Scale bar is 30 um.

To understand cell migration, multiple mechanisms have been proposed, such as actin polymerization, treadmilling, retrograde actin network flow, and myosin II-based contractility. All these mechanisms involve two cytoskeleton components, F-actin and myosin II. It has been reported that myosin II acts as a locomotion power in cell migration that fuels the assembling and disassembling of F-actin, which in turn provides the propelling force for cell migration. Therefore, we sought to determine whether matrix stiffness enhances pericyte invasiveness by promoting the assembling of F-actin and expression of myosin II. To this end, we dyed F-actin cytoskeleton and myosin II components of pericytes cultured on different matrix stiffness and visualized them under confocal microscope. In pericytes cultured on soft substrate (7.43 kPa), the actin structure did not organize into stress fibers but instead diffused evenly as cortical actin (Figure 4A). In contrast, on substrate of medium stiffness (16.7 kPa), pericytes started to exhibit orderly and nicely bundled actin structures which are typical of stress fibers. In the leading edges of the stress fibers, focal adhesions enriched with F-actin were clearly visualized (Figure 4A), which indicates that the adhesion force of pericytes increased as the matrix stiffens, for no focal adhesion plaque was observed when pericytes were cultured on soft substrate. On stiff substrates (34.88 kPa), F-actin cytoskeleton assembled into stress fibers that were well-stretched and more developed than those on soft and medium stiffness gels. Thus, the compliance of the substrate significantly affects the organization of the actin cytoskeleton. In contrast to F-actin, myosin II in pericytes did not seem to be affected by the matrix stiffness (Figure 4B), as myosin II in pericytes on different substrates remains steady and evenly scattered in cytoplasm, though the distributing area varies. Taken together, these data demonstrate that matrix stiffness may enhance pericyte invasiveness by increasing F-actin assembling and focal adhesion formation.

### Matrix Stiffness Induces Pericyte-Fibroblast Transition

**FIGURE 5:**
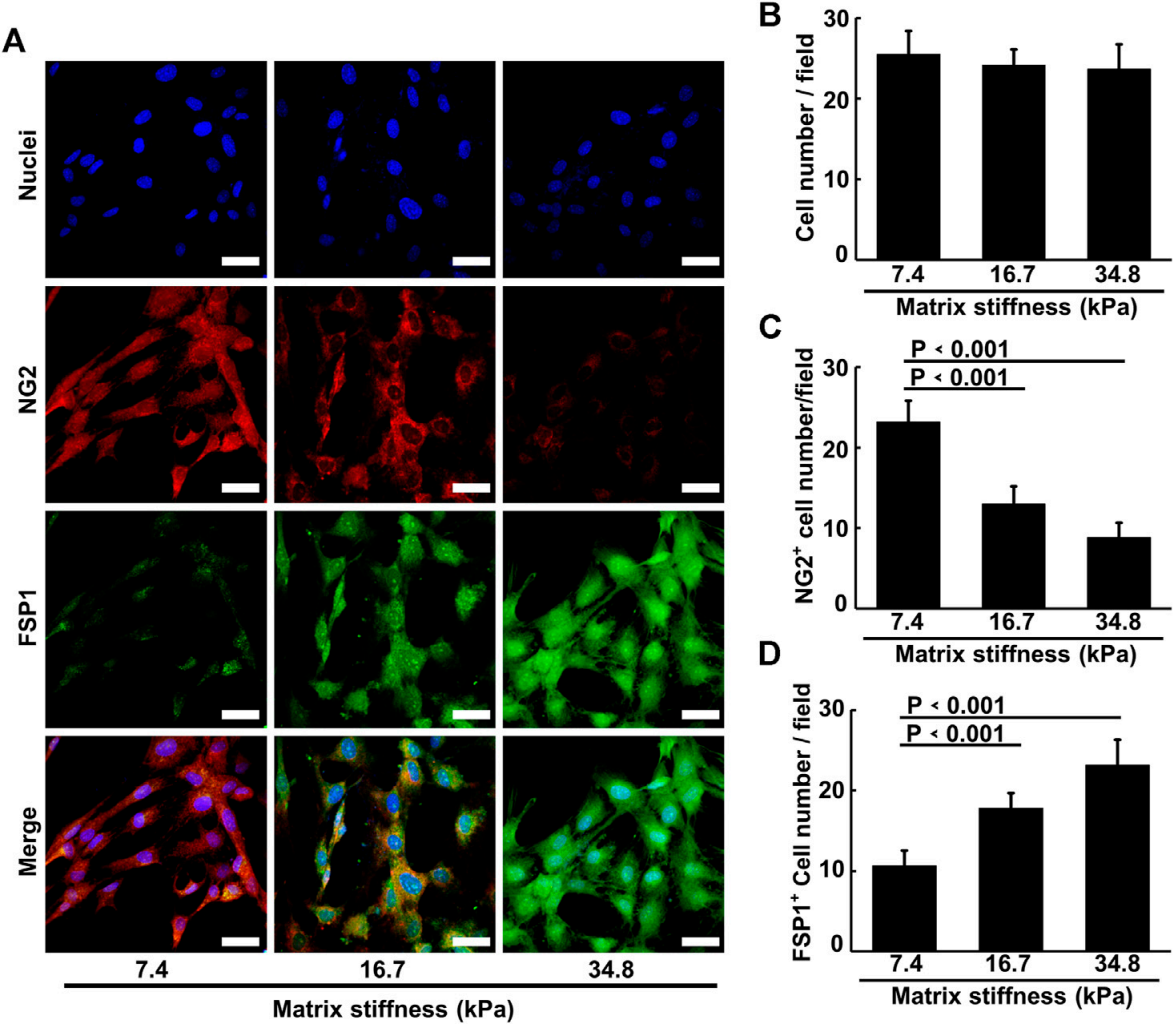
Matrix stiffness induces pericyte-fibroblast transition. **(A)** Representative immunofluorescence images of pericytes on different matrices stained with NG2 and FSP1 antibody. Cell nuclei were stained with DAPI. Scale bar is 20 um. **(B)** Statistical analysis of pericyte cell numbers in each image field. **(C)** Statistical analysis of NG2 positive pericyte cell numbers in each field. **(D)** Statistical analysis of FSP1 positive pericyte cell numbers in each field.

**FIGURE 6:**
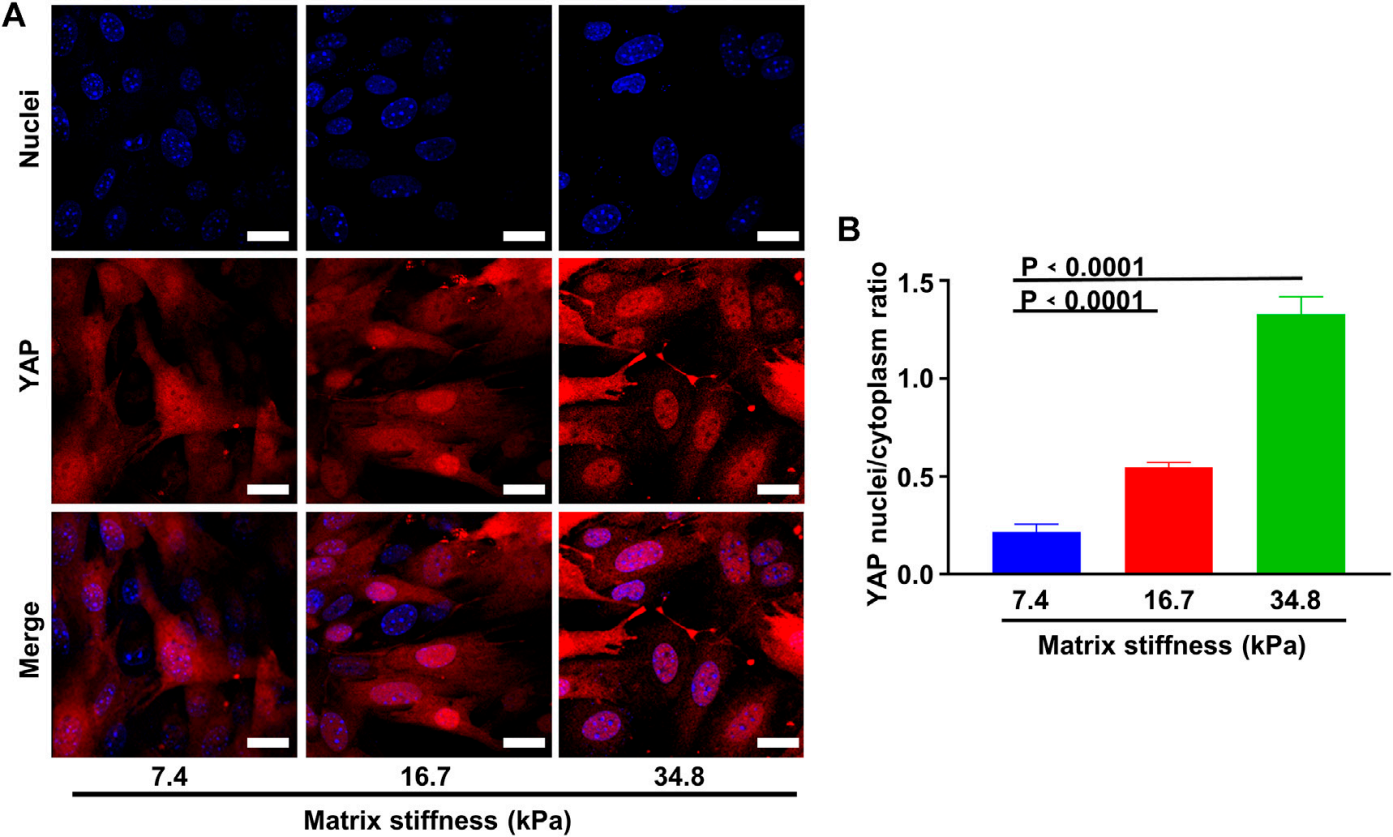
Matrix stiffness induces YAP activity. **(A)** Representative images of YAP activation along different matrix stiffness. Nuclei were stained with DAPI. Scale bar is 20 um. **(B)** Statistical analysis of YAP nuclei/cytoplasm ratio along different matrix stiffness.

The morphology changes and the invasive behavior pericytes gradually obtained along matrix stiffness suggested that pericytes might undergo differentiation into fibroblasts. To investigate this possibility, we cultured pericytes on different substrates for an extended period and conducted immunofluorescence experiment. As matrix stiffness increases, pericytes gradually lost the expression of NG2, and gained the expression of FSP1 (fibroblast-specific marker) (Figures 5A,C,D), indicating that pericytes underwent a pericyte-fibroblast transition, though the proliferation rate did not change much (Figure 5B). Multiple studies have reported a critical link between the extracellular mechanical environment and the intracellular signaling. Yes-associated protein (YAP) is a prominent transcriptional coactivator that translates extracellular physical information into protein expression by translocating to the nucleus and regulating messenger RNA expression. It has been reported that YAP mediate cellular mechano-responses and inhibition of YAP translocation inhibits mesenchymal stem cell differentiation (Dupont et al., 2011). In tumor tissue, matrix stiffening enhances YAP activation in fibroblast and the generation and maintenance of cancer-associated fibroblasts correlate strongly with YAP translocation. Therefore, we presumed that pericyte-fibroblast transition induced by matrix stiffness relies on the activation of the transcriptional activator YAP. To study the validity of this presumption, we stained pericytes cultured on different stiffness substrates and conducted confocal immunofluorescence experiments (Figure 6A). Pericytes cultured on soft substrate had most YAP in cytoplasm, but as substrate stiffness increases, the transcription coactivator YAP gradually activated and translocated into nucleus, as the ratio of nuclear YAP to cytoplasm YAP increased along the matrix stiffness (Figure 6B). Thus, we concluded that matrix stiffness induced pericyte-fibroblast transition and the transcription coactivator YAP was involved in this transition.

### Inhibition of YAP Translocation Prohibits Pericyte-Fibroblast Transition

**FIGURE 7:**
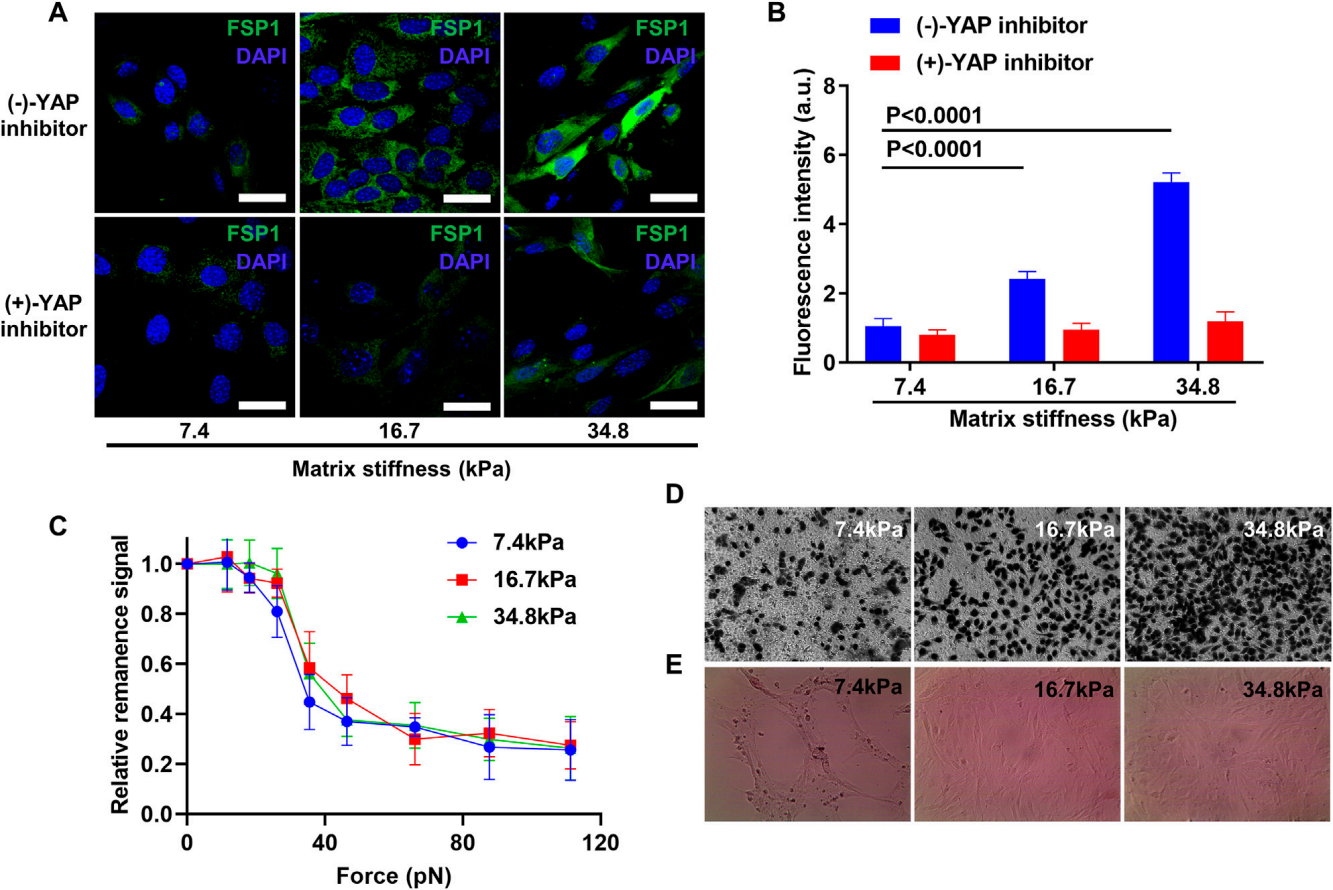
YAP activation inhibition prevents pericyte-fibroblast transition and abrogates adhesion enhancement of pericytes on stiff matrix. **(A)** Representative immunofluorescence images of pericytes stained with FSP1 and DAPI after YAP inhibition on different matrices. Scale bar is 30 um. **(B)** Statistical analysis of FSP1 fluorescence intensity in A. **(C)** FIRMs measurement of pericyte adhesion force after YAP inhibition on different matrices. **(D)** Representative Transwell images of mouse tumor cells after pericytes cultured on different matrices were seeded in Transwell chamber upon Matrigel but beneath mouse tumor cells. **(E)** *In vitro* angiogenesis of pericytes on different matrices.

Considering that YAP was activated in pericytes that were cultured on stiff substrates, and matrix stiffness induces pericyte-fibroblast transition; we asked if cell fates induced by stiff matrix require YAP activation and if the inhibition of YAP activation should prohibit pericyte-fibroblast transition induced by matrix stiffness. To answer this, we inhibited YAP translocation using verteporfin, a YAP inhibitor that inhibits the interaction between YAP and its nuclear combining partner TEAD. Verteporfin nearly completely abrogated the conversion of pericytes into fibroblasts, as indicated by the ground zero expression level of FSP1 in matrix stiffness-stimulated pericytes (Figures 7A,B). The expression of FSP1 in matrix stiffness-stimulated pericytes that were not subjected to verteporfin treatment remains an ascending profile, which were consistent with the matrix stiffness-induced pericyte-fibroblast transition finding. We further studied if the inhibition of YAP translocation could have any influence on pericyte adhesion pattern, as in tumor tissues alteration of pericyte adhesion pattern may indicate damage to the integrity of blood vessels. Inhibition of YAP activation overturned adhesion enhancement of pericytes stimulated by different matrix stiffness; the adhesion force of pericytes on different substrates did not wary significantly (Figure 7C). Based on these findings, we concluded that matrix stiffness induced pericyte-fibroblast transition through the activation of nuclear transcription factor YAP. Compared to pericytes, fibroblasts in tumor tissues are more aggressive. Tumor associated fibroblasts migrate away from tumor tissues and acting as trailblazers pave way for the dissemination of tumor cells, which tag along the migrating fibroblasts. To investigate if matrix stiffness induces the enhancement of tumor cell invasion by inducing pericyte-fibroblast transition, we seeded different matrix stiffness-stimulated pericytes into Matrigel-modified Transwell chamber, and used them as a new model to study tumor cell invasion. The results show that pericytes collected from soft matrix did not enhance the invasive behavior of tumor cells; however, pericytes collected from stiff matrix greatly increased the invasiveness of tumor cells (Figure 7D), which is in accordance with previous reports that in highly metastatic tumor tissues, where tumor metastasis often occurs through tumor blood vessel, the blood vessels are often leaky, tortuous and less covered by pericytes. To see if matrix stiffness could affect the integrity of blood vessels, we conducted *in vitro* angiogenesis. Soft matrix did not alter the formation of lumenal structures as in healthy body tissue; however, stiff matrix demolished the lumenal structures and disarrayed pericytes (Figure 7E), which indicates that matrix stiffness may disturb the formation of wholesome blood vessels by inducing pericyte-fibroblast transition and pericyte-converted fibroblasts aid tumor metastasis through the sabotaged blood vessels.

## Discussion

Pericytes are one of the major mural cells that protect blood vessel integrity. Loss of pericytes in tumor blood vessels has been correlated with tumor metastasis and poor prognosis (Cooke et al., 2012). Due to their particular location, pericytes are often subjected to cyclic mechanical strains. While these forces are essential in maintaining pericyte identity and physiology, a deviation in stimulative mechanical forces can often result in malicious health issues (Jaalouk and Lammerding, 2009). In this study, we demonstrate that matrix stiffness plays dual roles in modulating pericyte function. First, matrix stiffness increases the adhesion force of pericytes. This is partly due to the enlarged cell spreading areas and increased focal adhesion plaques in pericytes on stiff matrix. The assembling of stress fibers and increase in focal adhesion plaque numbers hint that pericytes may have undergone fundamental changes on stiff matrix. Second, matrix stiffness enhances pericyte invasiveness. The rise of matrix stiffness triggers pericytes to migrate away from their colonies and invade outer space. This observation, when reflected onto patients, may explain why tumor stiffening is often found accompanied by twisted blood vasculature that is leaky and pericyte deficient. The possible underlying mechanism that matrix stiffness prompt pericyte invasiveness is that pericytes are mechanical stimuli responsive; upon sensing mechanical changes that take place in the microenvironment, pericytes seek to adapt to the new environment by changing their own mechanical properties accordingly. However, this mechanism needs further exploration.

Further, we also reveal that pericytes are elastic progenitor cells that serve as a reservoir for fibroblasts. Upon receiving mechanical stimuli from the surrounding microenvironments, pericytes alter their identity and become active stromal fibroblast. Stromal fibroblasts are one of the major cellular components in solid tumors that are easily co-opted by cancer cells to perform tumor-development-promoting functions that are otherwise poorly efficient or altogether unavailable to the tumor (Kalluri and Zeisberg, 2006; Labernadie et al., 2017). While biochemical communication that promotes pericyte-fibroblast transition have been well studied, we identify a physical co-optive mechanism that involves matrix stiffening and the perception of mechanical stimuli by pericytes. Consistent with our findings, recent structural analysis also indicates that solid tumor tissues have lower pericyte coverage on their blood vessels than normal healthy tissues. However, the mechanism as to how matrix stiffness induces pericyte-fibroblast transition, whether it is matrix stiffness first enhance pericyte mechanical properties and then the enhanced mechanical properties cause pericyte identity alteration or the other way around has not been explored. This gap of knowledge might be attributable, in part, to the technical want of a working profile that could isolate mechanical properties from the cell per se. Taken together, these findings imply that: 1) pericytes are highly plastic, they adapt to different environment by switching between different identities; 2) soft environment as posed by vasculature endothelial cells contains the differentiation of pericytes; 3) proper mechanical stimuli triggers pericytes differentiation as mechanical stimuli did mesenchymal stem cells. In our system, matrix stiffness induces pericyte-fibroblast transition.

YAP is a downstream element in how cells perceive their physical microenvironment. The activation of YAP requires cell spreading and the formation of stress fiber and cytoskeletal tension (Ege et al., 2018). Cells read the mechanical stimuli of the microenvironment as levels of YAP activity, and manipulation of YAP levels can dictate cell behavior (Dupont et al., 2011; Yang et al., 2014). In this sense, the activation of YAP in pericytes on stiff matrix may be the cause of pericyte-fibroblast transition. It has been reported that YAP activity can override mechanical inputs, and decide the differentiation of mesenchymal stem cells (Rosado-Olivieri et al., 2019), which is in accordance with our findings. Besides the generation and maintenance of cancer-associated fibroblasts also require mechano-transduction and YAP-dependent matrix remodeling (Calvo et al., 2013), which corroborates our findings. However, if YAP activation alone could induce pericyte-fibroblast transition and maintain the identity of the converted fibroblasts have not been addressed.

In this work, we provide another example of how tumor cells manipulate the host cells for their own development. By growing in matrix stiffness, tumor cells co-opted pericytes to provide a way for tumor intravasation into tumor blood vessels. It has been reported that loss of pericytes make tumor blood vessels more susceptible for cancer cell intravasation and eventually metastasis (Hosaka et al., 2013). Loss of pericytes from tumor vessels may not only admit tumor cell intravasation and pericyte-fibroblast transition, but the converted fibroblast may also hijack tumor cells, and either guide tumor cells to intravasate or even together colonize distant organs to form initial metastatic niches. Indeed, it has been described that tumor cells carry their own fibroblasts as “soil” for them to “seed” and grow in distal organs (Fares et al., 2020). Thus, we concluded that upon matrix stiffness stimulation, pericytes play dynamic roles in cancer invasion and metastasis.

## Conclusion

In conclusion, our study reveals that upon matrix stiffness stimulation, pericytes enhance their mechanical properties, including invasiveness and adhesion, and undergo pericyte-fibroblast transition which results in the easier dissemination of tumor cells through blood vessels. Based on these findings, we propose that targeting pericytes may be an effective way to prevent tumor metastasis through blood vessels.

## Data Availability

The original contributions presented in the study are included in the article/Supplementary Material, further inquiries can be directed to the corresponding author.
