# Supplementary material for: Matrix Stiffness Induces Pericyte-Fibroblast Transition Through YAP Activation
Source: Front Pharmacol. 2021 May 31;12:698275. doi: 10.3389/fphar.2021.698275 (PMC8202079; doi:10.3389/fphar.2021.698275)
Supplement: Supplementary file 1 [file DataSheet1.docx]

Supplementary Material

# Supplementary Figures

**
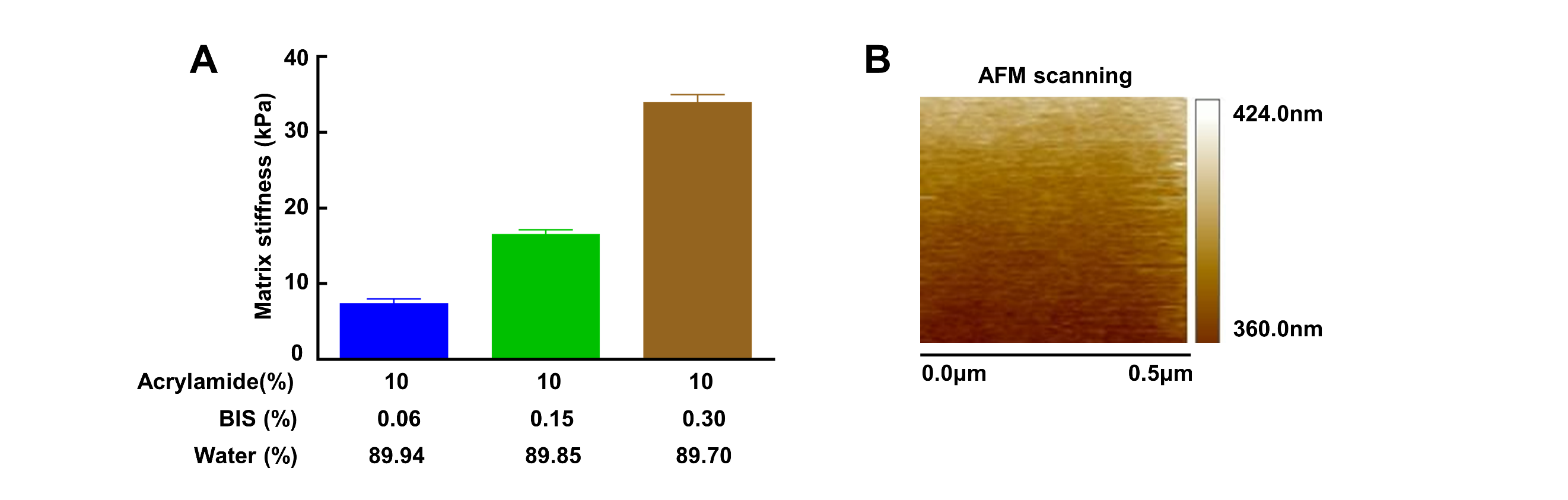
**

**Supplementary Figure 1.** Characterization of polyacrylamide (PA) gel substrates. (A) Characterization of PA gel matrix stiffness. (B) Representative images of surface morphology of the matrix scanned using AFM.


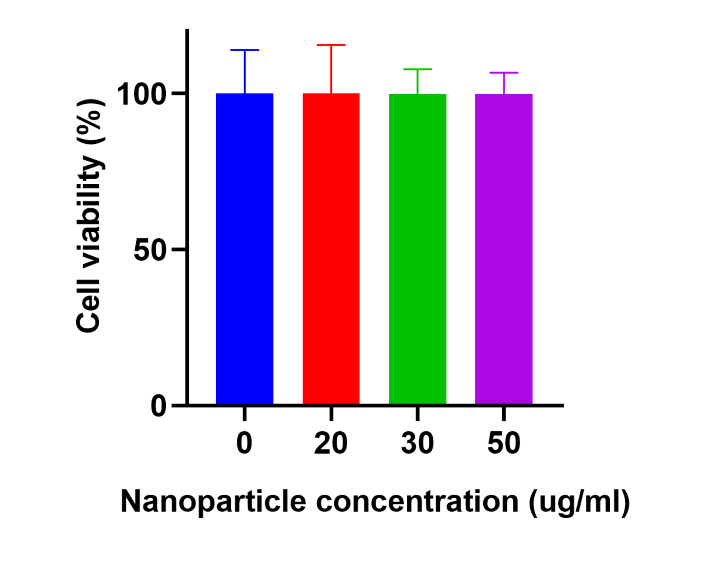


**Supplementary Figure 2.** Cytotoxicity of the nanoparticles. Data represented as mean ± SD.


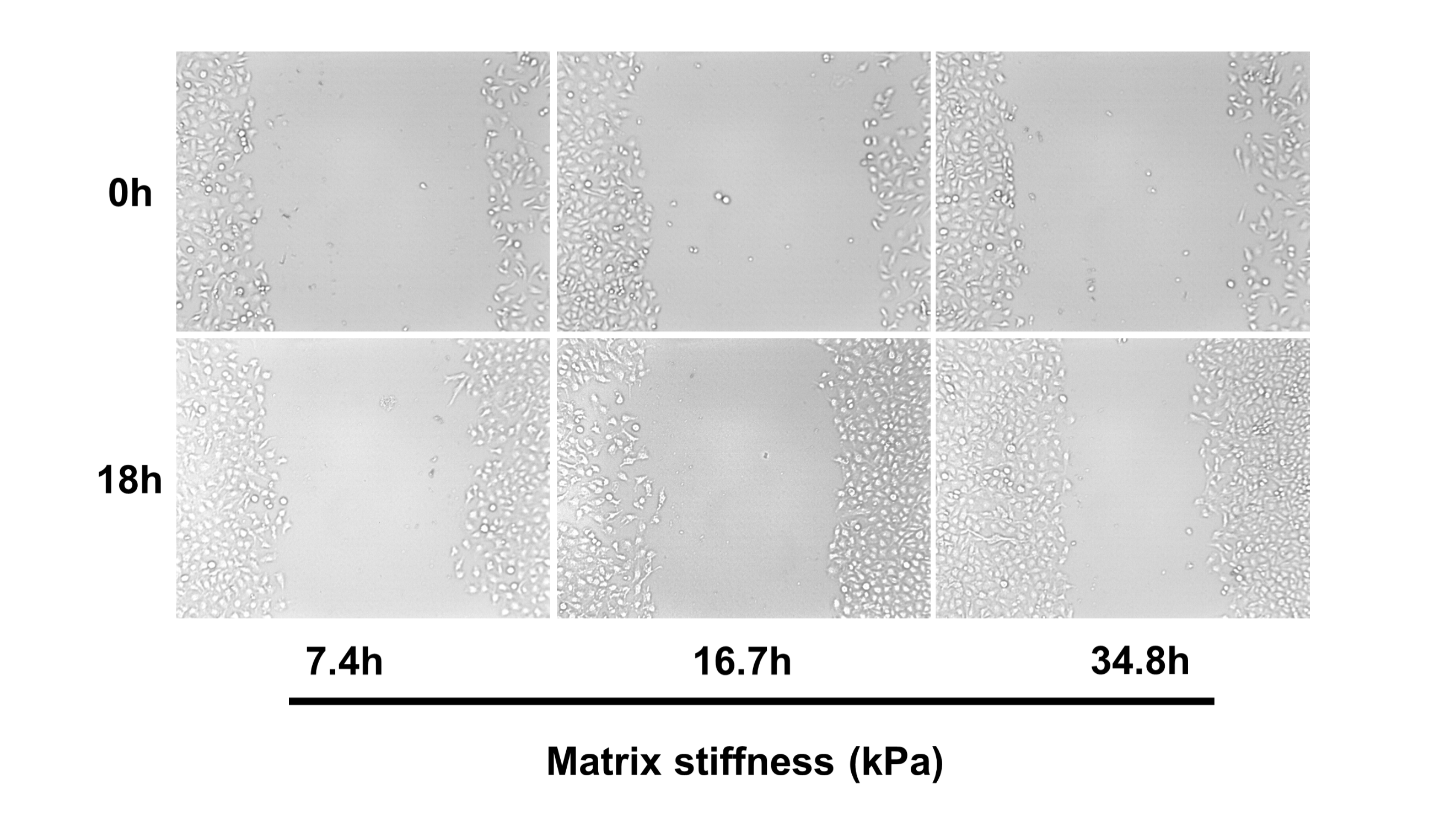


**Supplementary Figure 3.** Representative images of pericyte wound healing assay images.
